# Three-dimensional combined biomarkers assay could improve diagnostic accuracy for gastric cancer

**DOI:** 10.1038/s41598-017-12022-1

**Published:** 2017-09-14

**Authors:** Liping Sun, Huakang Tu, Tiejun Chen, Quan Yuan, Jingwei Liu, Nannan Dong, Yuan Yuan

**Affiliations:** 1 Tumor Etiology and Screening Department of Cancer Institute and General Surgery, The First Affiliated Hospital of China Medical University, Key Laboratory of Cancer Etiology and Prevention (China Medical University), Liaoning Provincial Education Department, Shenyang, Liaoning China; 2 Department of Epidemiology, The University of Texas MD Anderson Cancer Center, Houston, Texas USA; 3 Medical Oncology Department of Benxi Central Hospital, Benxi, China; 4 Cancer Hospital Chinese Academy of Medical Sciences, Shenzhen Center, Shenzhen, China

## Abstract

So far, stomach-specific biomarkers, gastric cancer(GC)-related environmental factors, and cancer-associated biomarkers are three major classes of serological biomarkers with GC warning potential, joint detection of which is expected to increase the diagnosis efficiency. We investigated whether the combination of serum pepsinogens(PGs), IgG anti-Helicobacter pylori (HpAb), and osteopontin (OPN) can be used as a panel for GC diagnose. Serum was collected from 365 GC patients and 729 healthy individuals,furtherly 332 cases and 332 age- and sex-matched controls were selected for the matched analysis. Serum levels were measured by ELISA. Logistic regression and receiver operator characteristic curve (ROC) were used to assess the associations of biomarkers with GC and the discriminative performance of biomarkers for GC. The area under ROC from three-dimensional combination of PGI/II-HpAb-OPN (0.826) was significantly higher than two-dimensional combination of PGI/II-HpAb (0.786, P < 0.001), PGI/II-OPN (0.787, P < 0.001), and OPN-HpAb (0.801, P = 0.006), as well as one-biomarker of PGI/II (0.735, P < 0.001), HpAb (0.737, P < 0.001) and OPN(0.713, P < 0.001), respectively. The combination of PGI/II-HpAb-OPN, yielded a sensitivity of 70.2% and specificity of 78.3% at the predicted probability of 0.493 as the optimal cutoff point. Three-dimensional combined biomarkers assay could improve diagnostic accuracy for gastric cancer.

## Introduction

More accurate biomarkers for prediction of gastric cancer (GC) may contribute for early detection to reduce the mortality. Currently available serological biomarkers for GC could be classified into three major categories: stomach-specific biomarkers, GC-related environmental factors and cancer-associated biomarkers, which have been investigated extensively for detecting GC.

These three types of biomarkers have distinguishing features. The former includes those specifically reflecting structure and function of the gastric mucosa (e.g., pepsinogens [PGs], gastrin-17 [G17] and TFFs), which could identify individuals with gastric mucosa alterations such as inflammation or atrophy, rather than GC itself^1,2^. Pepsinogen testing is a popular non-invasive method for GC screening, primarily in Japan and a few other countries^3–5^. As our previous studies and others have showed, PGI/II ratio (PGI/II) was more suitable for identifying gastric mucosa with atrophy or canceration compared with pepsinogen I(PGI) or pepsinogen II(PGII) alone^6,7^. GC-related environmental factors includes Helicobacter pylori (*H.pylori*) anti-IgG(HpAb) and Epstein-Barr virus (EBV), which could reflect the status of exogenous pathogenic factors infection. Chronic *H.pylori* infection-induced inflammation drives gastric carcinogenesis^8,9^. HpAb level may be reduced when atrophic gastritis spans most of the fundic area of the stomach after a prolonged infection of *H.pylori* ^10,11^. Our previous cross-sectional findings that serum anti-*H.pylori* IgG antibody titer was positively correlated with grade of histological gastritis and mucosal bacterial density^12^. The latter includes those closely related to the occurrence and progression of gastrointestinal cancers (e.g., carcinoembryonic antigen, CA 19-9, CA 72-4, E-cadherin, Mg7 and osteopontin (OPN)) which may be indicative of the presence of malignancy, but not specific of the organ from which the malignancy arises^13–15^. In our previous study, we found serum OPN levels, a multifunctional glycophosphoprotein, increased gradually when the gastric mucosa progressed from superficial gastritis to atrophic gastritis, and to GC^16^.

Although combining multiple independently predictive markers has been suggested to improve accuracy for detecting GC, however, to our knowledge, few study has investigated the diagnostic efficacy of three dimensional combined biomarkers for GC detection. In this study, we first jointly measured the expression of pepsinogen (PGI, PGII, PGI/PGII ratio), HpAb and OPN in serum, aiming to clarify whether the three-dimensional combined biomarkers assay could improve diagnostic accuracy for gastric cancer.

## Results

### The baseline serum level of biomarkers in the study participants

The baseline characteristics of the study participants and serum level of biomarkers were presented in Table 1. GC cases, on average, were older than the controls, and were more likely to be males. Serum levels of PGII, HpAb, and OPN were statistically significantly higher in the GC cases compared with the unmatched controls (P < 0.001) or the matched controls (P < 0.001). The PGI/II ratio (PGI/II) was statistically significantly lower in GC cases than that in the controls (P < 0.001). There was no significant difference in serum PGI level comparing GC cases with the unmatched controls (P = 0.422) or the matched controls (P = 0.788).

**Table 1:** Selected demographic characteristics and serum biomarker levels in GC and controls.

**Characteristics**	(A) Controls (n = 729)	(B) GC cases (n = 365)	(A) *vs*. (B) *P-*value	(C) Matched controls (n = 332)	(D) Matched cases (n = 332)	(C) *vs. (*D) *P-*value
Age (years, mean ± SD)	52.6 ± 10.3	60.0 ± 12.0	**<0.001**	57.9 ± 9.8	58.3 ± 11.1	0.635
**Gender**						
Male	338 (46.4)	256 (70.1)	**<0.001**	225 (67.8)	225 (67.8)	1.000
Female	391 (53.6)	109 (29.9)		107 (32.2)	107 (32.2)	
PGI (ng/mL)	92.8 ± 51.2	96.5 ± 80.3	0.422	97.6 ± 57.1	96.1 ± 81.7	0.788
PGII (ng/mL)	9.0 ± 10.4	16.9 ± 17.6	**<0.001**	10.6 ± 14.0	16.9 ± 17.9	**<0.001**
PGI/II	13.8 ± 8.6	8.2 ± 6.8	**<0.001**	12.5 ± 6.4	8.0 ± 5.8	**<0.001**
HpAb (EIU)	19.8 ± 22.6	46.0 ± 36.9	**<0.001**	19.2 ± 20.7	46.3 ± 36.5	**<0.001**
OPN (ng/mL)	2.0 ± 1.8	4.7 ± 4.0	**<0.001**	2.3 ± 2.1	4.7 ± 4.1	**<0.001**

### Associations of one-dimensional biomarker levels with gastric cancer

The subsequent comparative analyses were performed in matched cases-control groups. The association of one-dimensional biomarker with GC risk according to the quartiles of serum concentrations were presented in Table 2. Those in the lowest quartile of PGI relative to those in the highest quartile had statistically significant 5-fold higher odds of GC. Those in the highest quartile of the PGII relative to those in the lowest had statistically significant nearly 3-fold higher odds of GC. For the association of PGI/II with GC, the ORs for those in the first and second quartiles relative to those in the highest were statistically significant. For the association of HpAb with GC, the ORs for those in the third and fourth quartiles relative to those in the lowest, were statistically significant. For the association of OPN with GC, the ORs for those in the second, third, and fourth quartiles relative to those in the lowest, were, respectively, 1.42, 2.74, and 6.24, all of which except the first were statistically significant.

**Table 2:** Associations between baseline serum biomarkers levels (in quartiles) and GC risk.

Serum biomarkers	GC vs. control			
	GC (n)	Con (n)	OR (95% CI)	*P* ^a^ value
**PGI (ng/mL)**				
Quartile 1(0–64.1)	119	84	**5.12(1.81,14.45)**	0.002
Quartile 2(64.2–84.0)	65	82	1.60(0.53,4.84)	0.409
Quartile 3 (84.1–118.0)	56	84	0.86(0.26,2.88)	0.806
Quartile 4 (118.1–744.9)	91	82	Reference	N/A
**PGII (ng/mL)**				
Quartile 1(0–5.4)	64	86	Reference	N/A
Quartile 2(5.5–7.3)	36	84	0.58(0.35,0.96)	0.033
Quartile 3 (7.4–11.0)	50	80	0.84(0.52,1.36)	0.475
Quartile 4 (11.1–198.1)	181	82	**2.97(1.96,4.49)**	0.000
**PGI/II**				
Quartile 1(0–8.5)	216	83	9.17(5.41,15.54)	0.000
Quartile 2(8.6–11.6)	52	85	2.15(1.21,3.84)	0.009
Quartile 3 (11.7–15.8)	41	83	1.74(0.96,3.16)	0.068
Quartile 4 (15.9–42.0)	23	81	Reference	N/A
**HpAb (EIU)**				
Quartile 1(−0.6–4.3)	25	84	Reference	N/A
Quartile 2(4.4–12.1)	47	83	1.90(1.07,3.37)	0.216
Quartile 3 (12.2–28.1)	74	83	**3.00(1.74,5.17)**	0.000
Quartile 4 (28.2–180.3)	186	82	**7.62(4.55,12.78)**	0.000
**OPN (ng/mL)**				
Quartile 1(0–0.9)	31	87	Reference	N/A
Quartile 2(1.0–1.6)	41	81	**1.42(0.81,2.48)**	0.028
Quartile 3 (1.7–3.0)	82	84	**2.74(1.64,4.56)**	0.000
Quartile 4 (3.1–26.4)	178	80	**6.24(3.83,10.17)**	0.000

^*a*^ *P*: Compared with the N/A.

### Integrative diagnosis model for discriminating gastric cancer

**Figure 1:**
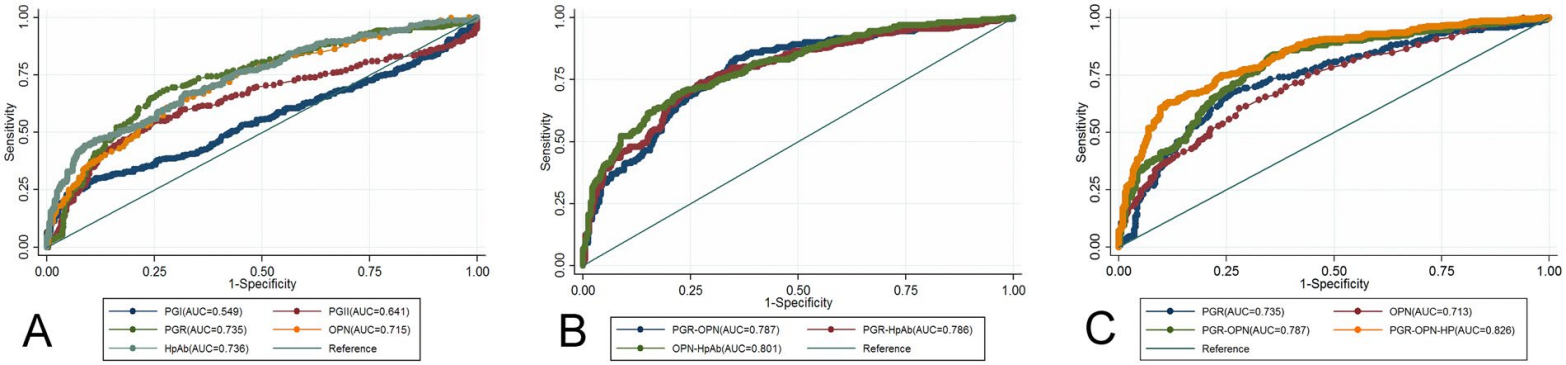
Receiver-operator characteristic curves of serum PGI, PGII, PGI/II(PGR), HpAb, and OPN individually and combined for discriminating between gastric cancer cases and controls. (**A**) Comparison of one-dimensional models; (**B**) Comparison of two-dimensional models; (**C**) Comparison of PGR-OPN-HP, PGR-OPN, PGR and OPN.

Figure 1 showed the receiver operator characteristic curve (ROC) analysis to assess the diagnostic efficiency of serum PGI, PGII, PGI/II, HpAb, and OPN, individually and combined, for discriminating between GC and controls.

As the one-dimensional models, PGI, PGII, PGI/II, HpAb and OPN, individually yielded area under the ROC curves(AUCs) of 0.550, 0.639, 0.735, 0.737, and 0.713, respectively. The biomarkers with an AUC greater than 0.7 (PGI/II, HpAb, and OPN) were further considered for combinations. Among the two-dimensional models (i.e., the models with two biomarkers), HpAb-OPN yielded the greatest AUC (0.801), followed by PGI/II-OPN (0.787) and PGI/II-HpAb (0.786). For the three-dimensional models (PGI/II-HpAb-OPN), the AUC (0.826) was greater than that of any of the one or two-dimensional models (P < 0.05) (Table 3).

**Table 3:** ROC analysis of serum biomarkers individually and combined for GC detection

**Models**		**AUC(95%CI)**	***P*** **-value**
Single-model	PGI	0.549(0.506–0.595)	
	PGII	0.641(0.596–0.682)	
	PGI/II	0.735(0.696–0.773)	
	HpAb	0.736(0.700–0.775)	
	OPN	0.715(0.675–0.752)	
Dual-model	PGI/II-OPN	0.787(0.752–0.822)	<0.001^a^, <0.001^b^
	PGI/II-HpAb	0.786(0.751–0.820)	<0.001^a^, <0.001^c^
	OPN- HpAb	0.801(0.768–0.834)	<0.001^b^, <0.001^c^
Tri-model	PGI/II-HpAb-OPN	0.826(0.795–0.858)	<0.001^d^, <0.001^e^,0.006^f^
	Lauren classification		
	intestinal	0.827(0.796–0.858)	0.219^g^
	diffuse	0.826(0.795–0.858)	
	Tumor stage		
	early	0.821(0.789–0.852)	0.469^h^
	late	0.816(0.784–0.848)	

^a^Compared with the AUC of PGI/II.

^b^Compared with the AUC of OPN.

^c^Compared with the AUC of HpAb.

^d^Compared with the AUC of PGI/II-OPN.

^e^Compared with the AUC of PGI/II-HpAb.

^f^Compared with the AUC of OPN- HpAb.

^g^Compared with the AUC between Lauren classification.

^h^Compared with the AUC between stage.

Furthermore, stratified analyses of the three-dimensional models were performed according to gender (male vs. female), age (<50 yrs vs. ≥50 yrs), major histopathological type (intestinal vs. diffuse) and tumor stage (early vs. advanced). There were no significant differences in the AUCs between strata (P = 0.902, P = 0.239, P = 0.219, P = 0.469, respectively). (see Table 3).

### Diagnostic performance of three-dimensional biomarkers for gastric cancer

For the one-dimensional models, according to the results of our previous research, the cutoff values were set 7.0 for PGI/II^6^, 34.0 EIU for HpAb [positive, Biohit], and 2.6 ng/mL for OPN^16^, respectively. For the multi-dimensional models, the optimal cutoff points were calculated based on the highest specificity with sensitivity >0.7. The corresponding diagnostic accuracy parameters including sensitivity, specificity, LR+ and LR− were shown in Table 4. The diagnostic performances of multi-dimensional model were more better than one-dimensional models: PGI/II yielded a sensitivity of 54.2% and specificity of 81%, HpAb yielded a sensitivity of 51.5% and specificity of 81%, OPN yielded a sensitivity of 64.2 and specificity of 67.5. PGI/II-HpAb yielded a sensitivity of 70.5% and specificity of 75.3% at the predicted probability of 0.472, OPN-HpAb yielded a sensitivity of 70.2% and specificity of 76.8% at the predicted probability of 0.462, and PGI/II-OPN yielded a sensitivity of 71.1% and specificity of 73.5% at the predicted probability of 0.495. Furtherly, the three-dimensional combination of PGI/II-HpAb-OPN yielded a sensitivity of 70.2% and specificity of 78.3% at the predicted probability of 0.493 as the optimal cutoff point.

**Table 4:** Accuracy of OPN, PGI/II, HpAb individually and combined for GC detection.

Biomarker	cutoff	Sensitivity (%)	Specificity (%)	LR+	LR−	Accuracy (%)	YD
PGI/II	7.0	54.2	81.0	2.9	0.7	56.5	0.352
HpAb (EIU)	34.0	51.5	81.0	2.7	0.6	66.3	0.325
OPN (ng/ml)	2.6	64.2	67.5	2.0	0.7	53.1	0.316
PGI/II- HpAb	0.472^a^	70.5	75.3	2.9	0.4	72.9	0.458
OPN- HpAb	0.462^a^	70.2	76.8	3.0	0.4	73.5	0.470
PGI/II-OPN	0.495^a^	71.1	73.5	2.7	0.4	72.3	0.446
PGI/II-OPN-HpAb	0.493^a^	70.2	78.3	3.2	0.4	74.3	0.485

^a^The cutoff value was selected as the diagnosis point of Sen >0.7 and the highest Sep.

## Discussion

The results from the present study suggested that the combination of serum PGI/II, HpAb and OPN had a stronger predictive ability for the presence of GC than any individual biomarker or any combination of two biomarkers did. At the predicted probability of 0.493 as the optimal cutoff point, the sensitivity was 70.2%, specificity was 78.3% and accuracy was 74.3% in our study population. To our knowledge, this is the first study to evaluate the three-dimensional combination of stomach-specific biomarkers, GC-related environmental factors, and cancer-associated biomarkers to predict the presence of GC.

PGs are product of terminally differentiated gastric mucosa. Human PGs have a diagnostic value for various gastroduodenal disorders, especially for peptic ulcer, atrophic gastritis and gastric cancer^17,18^. Based on currently available evidence, it has been proposed that serum levels of PGI, PGII, and PGI/II ratio might be useful for GC risk assessment^2,19,20^. However, a controversial and the most important weak point in the mass screening system by the PG method alone is the presence of the PG method-negative gastric cancer, especially, diffuse type gastric cancer.

*H.pylori* infection has been consistently associated with the risk of developing GC. It is well accepted that gastric adenocarcinoma, especially the intestinal type, is preceded by a prolonged *H.pylori*-driven precancerous process^21^. Numerous strands of evidence, including epidemiology, molecular studies, animal studies, and eradication studies in humans, had showed a reduced incidence of GC in those receiving eradication therapy. Eradication provided significant benefit for asymptomatic infected individuals and individuals after endoscopic resection of early GC. The overall GC risk reduction can be estimated at 34%^22^. But It is insufficient to check for *H.pylori* infection alone for the diagnosis of subjects with severe atrophic gastritis. As *H.pylori* directly induced PG release from isolated human peptic cells by nitric oxide and calcium-dependent mechanisms^23^, *H.pylori* infection correlated significantly with elevated serum PG, especially PGII, and reduced PGI/II ratio^24^. Therefore, the combination of serum anti-Hp IgG antibody and PG levels [ABC method] was proposed as a useful predictive marker for GC screening by Miki and some researchers since the early 1990s^4,25^. However, the low rate of secondary endoscopic examination following the ABC method is a negative factor for successful GC screening.

OPN is a secreted phosphorylated glycoprotein encoded by the SPP1 gene located on human chromosome 4q22.1. The major biological activities of OPN include regulating cell adhesion and chemotaxis, participating in cellular signal transduction, and stimulating a variety of downstream processes associated with cellular transformation or cancer progression^26–28^. Over the past decade, compelling evidence from experimental and clinical studies suggested that OPN overexpression was associated with clinic-pathological features and prognosis of GC^29–31^. Our previous research, which focused on investigating the relationships between serum OPN levels and risks of GC together with its influencing factors, has found serum OPN expression was closely related to GC risks, suggested that it might be a useful marker for the discrimination of GC^16^. However, as OPN is widely expressed in a variety of tissues, including bone, cartilage, kidney, blood vessels, skin and other tissue^32–35^, it itself lacks organ specificity and can’t be positioned.

In view of this, we have reason to believe that current single tests for GC provide relatively low sensitivity and specificity are possibly because the biomarkers are more single, not taking into account organ specificity, tumor specificity and exogenous specificity^36,37^. The use of multidimensional combination of serum biomarkers rather than a single one to identify individuals with GC risk can further improve specificity and sensitivity of the testing, resulting in higher sensitivity and specificity which improve diagnostic accuracy.

In this study, we firstly categorized PGI, PGII, HpAb and OPN concentrations according to quartiles to analysis the associations with GC risk. The results were shown that the lowest quartile of PGI, the highest quartile of the PGII, the first and second quartiles of PGI/II, the third and fourth quartiles of HpAb, and the second, third, and fourth quartiles of OPN were statistically significant between GC and controls. It confirmed that such three-dimensional biomarkers had potential value as a warning sign of GC. Furtherly, we evaluate the diagnostic performance of a three-dimensional biomarker panel for GC detection using AUC analysis. To sum up, combination of three-dimensional biomarkers improved the diagnostic accuracy for GC, with the sensitivity of 70.2% and specificity of 78.3%, which is far better than one-dimensional or two-dimensional biomarkers. As He *et al*. reported, the sensitivity of serum AFP, CEA, CA125 and CAl9-9 combination increased to 69.1%^38^. Another report showed that the sensitivity of the combination of serum CA72-4, CEA, CA125 and CA19-9 increased to 75.5%^39^. Furthermore, stratified analyses showed that the performance of the combination of PGI/II-HpAb-OPN did not differ significantly between Lauren classification (intestinal *vs*. diffuse), or Tumor stage (early *vs* late).

In addition, we selected 663 atrophic gastritis with intestinal metaplasia with 1:1 matched control on sex and age (within 5 years) for supplementary analysis. The results suggested that the combination of PGI/II and HpAb was better than any other single indicator or combination indicators. Therefore, the diagnostic performance of gastric cancer and precancerous diseases did have difference according to combination of categories. Three-dimensional combined biomarkers assay is more suitable for gastric cancer. Because the main purpose of this study was to investigate the application for gastric cancer diagnose, we showed those results in Supplementary Table 1 and Table 2.

Our study had several limitations. First, when selecting the study population, we did not investigate the proportion of patients having undergone *H.pylori* eradication. According to the cutoff ≥ 34 EIU (the cutoff value given by the test kit), the *H.pylori* positive rate in our subjects was 28.8%, which was relatively lower. So, we need further evaluate the availability of three-dimensional combined biomarkers assay in patients having received H.pylori eradication in order to determine its effective range of application. Second, we got the cutoffs for distinguishing GC only by ROC analysis. Although ROC can accurately reflect specificity and sensitivity, and the relationship between the test accuracy and representative, in the future, we would evaluate these predefined cutoffs in a validation follow-up cohort. Third, all patients with gastric cancer were preoperative diagnosis, some pathological information was incomplete (such as TNM stage, lymphatic metastasis, etc). However, this would not have affected our main outcome, multidimensional combination of serum biomarkers rather than a single one to identify individuals with GC risk can further improve specificity and sensitivity of the testing. Finally, our study population was limited to persons in a particularly high-risk region in northern China, so caution should be taken in generalizing our results to other populations. In addition, because the test panel have future potential to be implemented in clinical practice pending validation in clinical settings would be encouraged.

In summary, our results suggested that the combination of serum PGI/II, HpAb and OPN provided a certain extent accuracy for discrimination of GC, which performance was better than that of any single indicator. This three-dimensional panel of stomach-specific biomarkers, GC-related environmental factors and cancer-associated biomarkers, could be a potential application value tool for identifying patients who are at high risk of GC, especially to the regions with missing eradication programs or high prevalence of *H.pylori*. Future studies with a larger sample size are needed to confirm the predictive utility of this coin GC.

## Materials and Methods

### Study population

A total of 1094 participants (54% male, median age: 55 years, range 17–85 years) were recruited from Liaoning Province in China from 2002 to 2013. Among them, 683 participants were from the Zhuanghe Gastric Cancer Study, which is a population-based, combined serologic/endoscopic screening program for gastric diseases, conducted in Zhuanghe County. The study population selection and recruitment process has been previously reported^12,18^. Another 411 patients were histologically evaluated by routine outpatient elective gastric endoscopy with biopsies or surgical operation at the First Affiliated Hospital of China Medical University in Shenyang. Information on age, sex, and lifestyle (smoking and alcohol consumption) was obtained by interviewer-administered questionnaires. PPI should be discontinued at least 2 weeks before serum test. Antibiotics and bismuth compounds should be discontinued at least 4 weeks before the test. The following exclusion criteria were applied: i) previous upper gastrointestinal surgery or vagotomy; ii) ongoing treatment for any cancer; and iii) severe comorbidities, including hepatic, renal, cardiopulmonary, and hematologic disease.

On the whole, patients with GC were enrolled as cases (n = 365) prior to the resection. Participants with a histopathological diagnose of mild superficial gastritis were recruited as controls (n = 729). Furthermore, considering the influence of age and gender on serum levels of PG and OPN^6,16^, a subset of 332 GC cases was selected 1:1 matched control on sex and age (within 5 years) for subsequent comparative analysis.

The present study was approved by the Human Ethics Review Committee of the First Affiliated Hospital of China Medical University (Shenyang, China). Written informed consent was obtained from each participant.

### Histopathological assessment

Mucosal biopsies were obtained from the gastric body, angulus, antrum, and, if applicable, lesion site. Biopsies were processed by routine methods. Briefly, biopsies were oriented, fixed in 95% ethanol, embedded in paraffin blocks, and then sectioned and stained with hematoxylin and eosin at local study centers. Histopathological alterations of each stained section were independently evaluated by two gastrointestinal pathologists using standard criteria from the updated Sydney System for Gastritis^40^ and WHO classifications for GC^41^. Each participant was assigned a global diagnosis based on the most severe lesion found among all biopsy specimens.

According to Lauren’s classification, there were 106 intestinal type, 164 diffuse type and 31 mixed type. According to Tumor stage, there were 31 cases of early stage and 193 cased of advanced stage.

### Serological measurements

A 5-mL fasting venous blood sample was collected from each participant. All samples were centrifuged immediately at 3500 × *g* for 10 min, and a serum aliquot (400 µL) was frozen within 3 h after the blood drawn. Samples were stored at −80 °C until analysis. Serum levels of PGI, PGII, HpAb and OPN were measured by enzyme-linked immunosorbent assay (PGI enzyme-linked immunosorbent assay(ELISA), PG II ELISA and HpAb ELISA kits; BIOHIT Plc, Helsinki, Finland. OPN ELISA kit; Boster Biotechnology Company, Wuhan, China.) in the same aliquot and according to the manufacturer’s protocol blinded to the histopathological diagnosis. Duplicate negative and positive controls were included in each 96-well plate. Samples that yielded implausible values were re-tested. The mean intra-assay coefficients of variation were 11% for PGI, 13%for PGII, 11% for HpAb and 14% for OPN.

### Statistical analysis

Continuous variables were compared between cases and controls by the t-test and categories variables by the Chi-squared test. To estimate the predictive power of serum biomarkers individually or combined for GC, univariate and multivariate logistic regression analyses were performed. The odds ratios (OR) with 95% confidence intervals (95% CI) were calculated as measures of association using logistic regression analysis adjusted by age and sex. Receiver operator characteristic curves with corresponding C statistics (area under the curve, AUC) based on logistic models were used to determine the corresponding cutoff points when the pathologic diagnosis was treated as the “gold standard”, and measure the discriminatory performance of each biomarker or combination of biomarkers. Observations with missing data were excluded from analysis. All statistical analyses were performed using STATA version 13 (StataCorp, College Station, TX, USA). A *P* value ≤ 0.05 (two-sided) was considered statistically significant.

## Electronic supplementary material

Supplementary table 1;Supplementary table 2
