# Supplementary material for: Three-dimensional combined biomarkers assay could improve diagnostic accuracy for gastric cancer
Source: Sci Rep. 2017 Sep 14;7:11621. doi: 10.1038/s41598-017-12022-1 (PMC5599671; doi:10.1038/s41598-017-12022-1)
Supplement: Supplementary file 1 — Supplementary table 1;Supplementary table 2 [file 41598_2017_12022_MOESM1_ESM.pdf]

Three-dimensional combined biomarkers assay could improve diagnostic accuracy for gastric cancer

Liping Sun[1*], Huakang Tu[1,2*], Tiejun Chen [1,3], Quan Yuan [1,4], Jingwei Liu[1], Nannan Dong[1], Yuan Yuan[1#]

Supplementary table 1. Selected demographic characteristics and serum biomarker levels in atrophic gastritis and controls

factor	Controls （n=663）	Atrophic gastritis （n=663）	*P-value* [a]
Age (years, mean ± SD）	54.3±9.0	55.0±9.9	0.190
Gender			
Male	328(49.5%)	328(49.5%)	1.000
Female	335(50.5%)	335(50.5%)	
PGI (ng/mL)	94.1±51.9	102.4±59.2	**0.007**
PGII (ng/mL)	9.3±10.8	15.2±11.4	**<0.001**
PGI/II	13.3±6.8	8.9±6.1	**<0.001**
HpAb (EIU)	20±23.1	50.7±37.4	**<0.001**
OPN (ng/mL)	2.1±1.9	2.4±2.2	**0.002**

[a]*P-value*: atrophic gastritis vs controls.

Supplementary table 2. diagnostic efficacy of PGI/II, HpAb, OPN individually and combined for atrophic gastritis detection

Biomarker	cutoff	Sensitivity (%)	Specificity (%)	YD
PGI/II	7.0	48.3	85.3	0.336
HpAb (EIU)	33.9	59.3	81.9	0.412
OPN (ng/ml)	1.2	70.2	42.4	0.126
PGI/II- HpAb	0.477	70.0	75.7	0.457
OPN- HpAb	0.392	70.0	69.1	0.391
PGI/II-OPN	0.407	70.0	66.2	0.362
PGI/II-OPN-HpAb	0.411	70.0	72.9	0.429

[a]The cutoff value was selected as the diagnosis point of Sen>70% and the highest Sep.
